# Risk factors associated with mortality in neonatal intrahepatic cholestasis caused by citrin deficiency (NICCD) and clinical implications

**DOI:** 10.1186/s12887-018-1383-5

**Published:** 2019-01-14

**Authors:** Kuerbanjiang Abuduxikuer, Rui Chen, Zhong-Lin Wang, Jian-She Wang

**Affiliations:** 1 Department of Hepatology, Children’s Hospital of Fudan University, 399 Wanyuan Road, Shanghai, 201102 China; 2 Department of Infectious Diseases, Children’s Hospital of Fudan University, 399 Wanyuan Road, Shanghai, 201102 China

**Keywords:** Neonatal intrahepatic cholestasis caused by citrin deficiency (NICCD), infant, Mortality, Risk factors

## Abstract

**Background:**

Neonatal intrahepatic cholestasis caused by citrin deficiency (NICCD) has high prevalence in East Asia, and has been reported in other parts of the world. NICCD is also the most common form of genetic cholestasis among East Asians. There has been reports of mortalities or liver transplants associated with NICCD, but risk factors associated with poor outcome were unknown. Our objective is to report NICCD mortalities in a tertiary pediatric hepatology center, and to explore associated risk factors along with implications to clinical practice.

**Method:**

This is a retrospective analysis of NICCD cases collected from June 2003 until January 2017 in the Children’s Hospital of Fudan University. Clinical, biochemical, and genetic data were compared between deceased cases and survivors without liver transplant.

**Results:**

Sixty-one confirmed NICCD cases, including 52 cases in the survival group, and 9 cases in the mortality group, were included in the analysis. Mean age at referral in the mortality group was significantly higher when compared to the survival group (9.58 ± 5.03 VS 3.96 ± 3.13 months, *p* < 0.000). The proportion with infection in the mortality group was significantly higher than the survival group (*p* = 0.023). 44.4% of patients in the mortality group did not receive lactose-free and/or medium chain triglycerides enriched (LF/MCT) formula, and this percentage was significantly higher than the survival group (9.6%, *p* = 0.021). Mean platelet (PLT) count in the mortality group was significantly lower than the survival group (*p* = 0.010). Mean serum gamma-glutamyl transpeptidase (GGT), and total cholesterol (TCH) levels were significantly lower in the mortality group when compared to the survival group with *p* values of 0.001, and 0.019, respectively. Those who died had higher serum ammonium levels than survivors (*p* = 0.016). Mean level of citrulline was significantly lower in the mortality group compared to the survival group (*p* = 0.010). On the other hand, mean level of tyrosine was significantly higher in the mortality group than that of the survival group (*p* = 0.015).

**Conclusion:**

Late referral, presence of infection, delayed treatment with LF/MCT formula, lower platelet count, lower levels of GGT, total cholesterol, blood citrulline, and higher level of blood ammonia and tyrosine, were associated with poor prognosis in NICCD.

**Electronic supplementary material:**

The online version of this article (10.1186/s12887-018-1383-5) contains supplementary material, which is available to authorized users.

## Background

Citrin deficiency is an autosomal recessive disorder caused by mutations in the *SLC25A13* (solute carrier family 25 member 13) gene. Three main phenotypes, including neonatal intrahepatic cholestasis caused by citrin deficiency (NICCD) during infancy, failure to thrive and dyslipidemia caused by citrin deficiency (FTTDCD) in older children, and recurrent hyperammonemia with neuropsychiatric symptoms as citrullinemia type II (CTLN2) in adults, have been recognized [1]. Citrin deficiency was first reported in Japan [2], but later was recognized as a worldwide disease with high prevalence in East-Asian countries [3]. Carrier frequency of pathogenic *SLC25A13* gene variant is the highest in southern China including Taiwan (1:48), followed by Japan (1:65) [4, 5], Korea (1:115), and northern China (1:940) [6, 7]. The observed prevalence of NICCD in Japan is similar to calculated homozygous and compound heterozygous carrier rate (1:17000) [1, 8], and over 80,000 East Asians are estimated to be homozygous for *SLC25A13* gene pathogenic variants [6]. Caused by biallelic mutations in the *SLC25A13* gene, NICCD is also the most common form of genetic cholestasis among East Asians. NICCD usually present itself as neonatal cholestasis, and characterized by decreased alanine aminotransferase (ALT) to aspartic acid transaminase (AST) ratio, hypoglycemia, decreased albumin level, prothrombin time (PT) elongation, multiple amino acidemia, high levels of alpha-fetoprotein (AFP), and fatty liver [1, 9]. NICCD is usually regarded as a benign process that resolve spontaneously or after administration of lactose-free and/or medium-chain triglycerides enriched (LF/MCT) formula [9, 10]. However, there were 11 case reports or individuals from case series from eight centers in English literature who needed liver transplantation for liver failure or died before the transplantation took place because of NICCD [10–18]. Moreover, clinical, biochemical, and genetic characteristics of already reported cases with poor outcome were not clearly outlined, and risk factors associated with death or liver transplant were unknown. Due to high prevalence of NICCD in East Asia and evidence of affected cases in other parts of the world, there is a need to explore risk factors that could lead to poor prognosis. Here we report nine cases of NICCD mortality from a tertiary pediatric hepatology center in China, present clinical, laboratory, and genetic features, and explored associated risk factors. To date, this is the largest number of NICCD mortality ever reported from a single center with detailed description of clinical, laboratory, and genetic features, and first analysis of risk factors associated with poor prognosis. We also discussed implications to clinical practice, and strategies for improving prognosis.

## Methods

### Subjects

We collected patients referred to the Department of Hepatology in the Children’s Hospital of Fudan University (Shanghai, China) for investigation of cholestasis with disease onset before six-months of age between June 2003 and January 2017. All patients were screened for *SLC25A13* gene mutations, and the screening process was previously published elsewhere [7]. Patients with homozygous or compound heterozygous disease-causing mutations were diagnosed to have NICCD and directly enrolled into this study. When SLC25A13 genetic analysis yielded a single heterozygous mutation, western blot analyses of citrin protein were performed using liver or skin biopsy specimen to confirm the absence of citrin protein before diagnosing NICCD [19]. Patients with single heterozygous mutation but have normal expression of citrin protein in western blotting of liver and/or skin samples were excluded as with confirmed cases with insufficient data. The Ethics Committee in Children’s Hospital of Fudan University waived ethics approval for using medial and genetic data of patients included in this cohort to be used for retrospective analyses. Authors participated in this study have no competing interests to declare.

### Methods

The medical records of all included cases were reviewed and abstracted. The guardians of every case were contacted by telephone, email, or regular mail to get the up-to-date information about prognosis. Gender, birth weight, *SLC25A13* gene mutation, age at referral, serum biochemistry at presentation, blood coagulation panel, complete blood count, tandem mass spectrometry, and clinical management were compared between deceased cases and survivors without liver transplantation. Original dataset with variant description used for statistical analyses was provided as an Additional file 1.

STATA software (version 12.0 Special Edition, STATA Corp, College Station, TX) was used for statistical analysis. Chi square test was used for categorical data, and Fisher’s exact values were calculated when expected values were five or less. Continuous variables were presented as mean ± standard deviation (SD), and Shapiro–Wilk normality test was performed to determine if each continuous variable is normally distributed. Normally distributed continuous variables were compared by using Student’s t-test, while non-parametric Wilcoxon-Mann-Whitney tests were performed to compare variables that were not normally distributed. A two-sided *P* values of less than 0.05 were regarded as statistically significant.

## Results

### General information

64 NICCD cases were confirmed genetically and/or with Western blot analysis. Among of them, 56 cases were genetically confirmed, including 21 cases with homozygous mutation, and 35 cases with compound heterozygous mutation in SLC25A13 gene. Eight cases with single heterozygous mutation were confirmed by absence of citrin protein by western blot analyses on liver sample (4 cases) or skin fibroblast (4 cases). Apart from one case born to fourth-generation cousins, no consanguineous marriages were found from parents of other cases. Up to the time of preparation of this manuscript, 54 patients have survived without liver transplant, nine cases have died, and 1 lost to follow-up. After excluding 1 case that lost to follow-up and 2 infants with insufficient follow-up data, 61 confirmed NICCD cases, including 52 cases (30 males, and 22 females) with available data in the survival group, and nine cases (seven males, and two females) in the mortality group, were included in the final analysis.

*SLC25A13* gene mutation, age at referral, diagnosis, administration of LF/MCT formula, and age of death in nine deceased cases were recorded in Table 1. All cases with poor prognosis had homozygous or compound heterozygous deleterious mutations such as insertion/deletion or splice site mutations. Most cases (seven out of nine) were referred to our center after 6-months of age, while two cases presented after 1-year of age. LF/MCT formula was started after referral in 5 cases, 4 cases did not respond to dietary change, but one case suffered from unexplained death after infection while liver function was improving. Four cases did not receive LF/MCT formula due to non-adherence or atypical presentation. The youngest age of death was five-months, while the oldest child died at the age of 23 months. Eight cases died from liver failure, while one case suffered from unexplained death after infection. Five cases had evidence of infection prior to death, one infant had concomitant kidney failure, while another child had interstitial lung disease and brain MRI abnormality.

**Table 1:** Characteristics of deceased cases

No	SLC25A13 gene mutations	Age at referral (months)	Condition at referral	LF/MCT formula	Age of death (Mo)	Cause of death
1	851del4/1638ins23	6	Cirrhosis, liver failure	No	8	Liver failure
2	851del4/IVS16ins3kb	9	Liver failure, hepatosplenomegaly, bile sludge, ascites	No	9.5	Liver failure
3	851del4/IVS16ins3kb	5	Liver failure	No	6	Liver failure
4	851del4/851del4	4	cholestasis	Yes	5	Infection, sudden death
5	851del4/851del4	19	cholestasis	Yes	23	Liver failure, infection
6	851del4/IVS6 + 5G > A	6	cholestasis	Yes	9	Liver failure, infection
7	851del4/IVS16ins3kb	11	Liver failure, hepato-renal syndrome	No	11	Liver failure, kidney failure
8	851del4/1638ins23	6	cholestasis	Yes	6	Liver failure, recurrent infection
9	851del4/1638ins23	13	Liver failure, hepatosplenomegaly,	Yes	13	Liver failure, diarrhea, suspected interstitial lung disease, brain MRI abnormality

### Risk factors associated with mortality

Sixty-one cases, including 52 children with available data in the survival group without liver transplantation, and 9 cases in the mortality group, were included in the final analysis. Gender, birth weight, age at referral, and blood test results at referral (complete blood count, serum biochemistry, blood coagulation profile, tandem mass spectrometry, and genetic test results) were compared to explore the risk factors associated with NICCD mortality (Table 2).

**Table 2:** Comparison of clinical and laboratory data between survival group and mortality group

Patient characteristics (Reference range)		Survival group (*n* = 52)	Mortality group (*n* = 9)	*P* value
General Information	Gender (Male/Female)	30/22	7/2	0.462
	Birth weight (g)	3079.32 ± 694.17(44)	2828.57 ± 360.39(7)	0.351
	Age at referral (months)	3.96 ± 3.13(48)	9.58 ± 5.03(8)	**0.000**
	Presence/absence of infection (n)	29 /21	7/1	**0.023**
	Lactose-free and/or MCT-enriched formula (Yes/No)	47/5	5/4	**0.021**
Complete blood count	WBC (4–10 *10^9/L)	10.34 ± 4.91(30)	9.90 ± 2.7(5)	0.637
	RBC (3.5–5.5 *10^12/L)	3.34 ± 0.66(23)	2.94 ± 0.87(5)	0.255
	PLT (100–300 *10^9/L)	387.54 ± 196.46(28)	109.60 ± 19.26(5)	**0.010**
	Hemoglobin (110–160 g/L)	93.81 ± 22.40(29)	87.40 ± 22.63 (5)	0.342
Serum Biochemistry	Total Biliburin (5.1–17.1 umol/L)	117.86 ± 65.73(47)	195.10 ± 194.99(8)	0.503
	Direct Bilirubin (0–6 umol/L)	70.82 ± 39.48(47)	113.69 ± 99.46(8)	0.474
	ALT (0–40 IU/L)	44.51 ± 71.35(47)	45.25 ± 25.44(8)	0.148
	AST (0–40 IU/L)	98.41 ± 96.59(46)	100.75 ± 60.47(8)	0.450
	GGT (7–50 IU/L)	219.32 ± 127.59(47)	80.00 ± 69.70(8)	**0.001**
	GGT (<=50 vs. > 50 IU/L)	1/46	4/4	**0.001**
	Total bile acid (0–10 ummol/L)	185.47 ± 84.47(46)	140.88 ± 124.80(8)	0.206
	Total protein (60–83 g/L)	49.45 ± 9.56(45)	49.84 ± 6.38(8)	0.914
	Albumin (35–55 g/L)	32.74 ± 9.93(45)	30.89 ± 6.05(8)	0.205
	Glucose (3.9–5.8 mmol/L)	2.84 ± 1.28(46)	3.36 ± 1.38(8)	0.318
	Total cholesterol (3.1–5.2 mmol/L)	3.30 ± 1.06(41)	2.25 ± 1.03(7)	**0.019**
	Triglyceride (0.56–1.70 mmol/L)	1.48 ± 0.65(40)	1.39 ± 0.69(8)	0.571
	Urea (2.5–6.5 mmol/L)	4.02 ± 4.27(27)	2.91 ± 1.10(5)	0.815
	Creatinine (20–110 umol/L)	20.28 ± 14.00(29)	15.51 ± 5.51(8)	0.271
	Lactic acid (0.7–2.1 mmol/L)	3.63 ± 2.76(15)	3.98 ± 1.22(5)	0.708
	Serum ammonia (10–47 umol/L)	98.14 ± 45.20(34)	142.31 ± 42.09(8)	**0.016**
	Alphafetoprotein (0–28 ng/ml)	15,473.97 ± 25,750.21(26)	60,476.24 ± 126,197.60(5)	0.823
Blood Coagulation Profile	INR (0.8–1.2)	1.41 ± 0.38(34)	1.60 ± 0.58 (7)	0.510
	PT (12.0–14.8 s)	17.51 ± 6.35 (37)	18.37 ± 6.18 (7)	0.712
	PTA (80–120%)	71.75 ± 25.75 (28)	68.57 ± 34.40 (7)	0.786
	APTT (28.0–44.5 s)	45.63 ± 13.29 (37)	53.09 ± 12.56 (7)	0.177
	Fib (2–4 g/L)	2.04 ± 3.34 (32)	1.74 ± 1.05 (7)	0.656
	Thrombin Time (14–21 s)	21.26 ± 4.15 (38)	21.66 ± 3.66 (7)	0.817
Blood tandem mass spectrometry	Citrulline (7–40 umol/L)	137.06 ± 79.62 (31)	52.34 ± 19.14 (4)	**0.010**
	Methionine (10–80 umol/L)	157.26 ± 100.04 (23)	231.93 ± 324.79 (5)	0.787
	Tyrosine (30–200 umol/L)	130.00 ± 73.19 (21)	250.26 ± 77.78 (3)	**0.015**
	Threonine (17–90 umol/L)	130.67 ± 51.62 (20)	228.30 ± 212.56 (2)	0.732
SLC25A13 gene mutation allele frequency	851del4	55 (104)	11 (18)	0.518
	1638ins23	12 (104)	3 (18)	0.541
	IVS6 + 5G > A	6 (104)	1 (18)	1.000
	IVS16ins3kb	6 (104)	3 (18)	0.128

Boldface, statistically significant *p* values

Distribution of gender and mean birth weight were not significantly different between the survival group and the mortality group (*p* values were 0.462 and 0.351, respectively). On the other hand, mean age at referral in the mortality group (9.58 ± 5.03 months) was significantly higher when compared to the survival group (3.96 ± 3.13 months, *p* < 0.000). Significantly more children (87.5%) in the mortality group had infection when compared to the survival group (58.0%, *p* = 0.023). 44% (4/9) of patients in the mortality group did not receive lactose-free and/or MCT-enriched formula, and this percentage was significantly higher than that of the survival group (9.6%, *p* = 0.021).

White blood cell (WBC) count, red blood cell (RBC) count, and hemoglobin levels were similar between the survival group and the mortality group (*p* values were 0.637, 0.255, and 0.342, respectively). However, PLT in the mortality group (109.60 ± 19.26*10^9/L) was significantly lower than that of the survival group (387.54 ± 196.46*10^9/L, *p* = 0.010).

Mean serum GGT level in the mortality group (87.43 ± 71.78 IU/L) was significantly lower than that of the survival group (223.37 ± 125.91 IU/L, *p* = 0.001). Mean serum Total cholesterol level in the mortality group (2.12 ± 1.19 mmol/) was significantly reduced in the mortality group, while remained within normal range (3.47 ± 0.97 mmol/L, *p* = 0.012) in patients from the survival group. Children with poor prognosis had similar total bilirubin (195.10 ± 194.99 umol/L) and direct bilirubin (113.69 ± 99.46 umol/L) levels when compared to those who survived (117.86 ± 65.73, and 70.82 ± 39.48 umol/L, *p* values were 0.503 and 0.474, respectively). Significantly more patients in the morality group had normal serum GGT levels when compared to the survival group (4/8 VS 1/47, *p* = 0.001). On the other hand, mean serum ammonia level in deceased children (142.31 ± 42.09 mmol/L) was significantly higher than those who survived without liver transplant (98.14 ± 45.20 mmol/L, *p* = 0.016). Other biochemical parameters, including alanine aminotransferase (ALT), aspartate aminotransferase (AST), total bile acid, total protein, albumin, blood glucose, serum triglyceride, urea, creatinine, lactic acid, and alpha-fetoprotein levels were similar between two groups (*p* values were 0.148, 0.450, 0.206, 0.914, 0.205, 0.318, 0.571, 0.815, 0.271, 0.708, and 0.823, respectively).

Blood coagulation profiles, such as international normalized ratio (INR), prothrombin time (PT), prothrombin activity (PTA), activated partial thromboplastin time (APTT), fibrinogen (Fib), and thrombin time (TT) were all similar when compared between the survival group and the mortality group (*p* values were 0.510, 0.712, 0.786, 0.177, 0.656, and 0.817, respectively).

Blood amino-acid profiles on tandem mass spectrometry were compared between two groups. Mean level of citrulline in the mortality group was 52.34 ± 19.14 umol/L, and was significantly lower in when compared to the survival group (137.06 ± 79.62 umol/L, *p* = 0.010). On the contrary, mean level of tyrosine in the mortality group was 250.26 ± 77.78 umol/L, significantly higher than that of the survival group (130.00 ± 73.19 umol/L, *p* = 0.015). As for methionine and threonine levels, there were no statistically significant differences between two groups (*p* values were 0.787, and 0.072, respectively).

*SLC25A13* gene mutation allele frequencies, including 851del4, 1638ins23, IVS6 + 5G > A, and IVS16ins3kb, were compared between 2 groups. However, none of these mutation allele frequencies were different between the morality group and the survival group (p values were 0.518, 0.541, 1.000, and 0.128, respectively).

## Discussion

NICCD is an autosomal recessive urea cycle disorder commonly occurred among East-Asians. It is caused by *SLC25A13* gene mutation, and usually regarded as self-limiting disease in which clinical and laboratory abnormalities begin to improve after complementary feeding. Changing to LF/MCT formula during infancy may facilitate the healing process, or even reverse the liver damage [1, 9].

The first case report of poor outcome associated with NICCD was in 2002 by Tamamori et al. from Japan [11]. A 7-month-old infant with neonatal cholestasis, whom initially diagnosed to have tyrosinemia type 1 received liver transplant at the age of 10 months due to liver failure. Post-transplant sequencing confirmed compound heterozygous mutations of 851del4/IVS11 + 1G in *SLC25A13* gene. There were 2 more cases of NICCD with high tyrosine levels by Shigeta [12] and Ohura [10], and both received liver transplant. The third case in our series was also suspected to have tyrosinemia but died without response to low-protein-diet. Screening for blood samples for common mutations in SLC25A13 gene found compound heterozygous 851del4/IVS16ins3kb mutations. Mean blood tyrosine level in mortality group in our series was significantly higher than those who survived. The transplanted NICCD case report by Shigeta et al. [12] had normal levels of citrulline. Mean citrulline level in our deceased case series was significantly lower when compared to those with good prognosis. Protein restriction and hypoglycemia may have led to higher carbohydrate intake, more intravenous glucose supplementation, and delayed lactose restriction in these patients that could lead to further metabolic derangements [20, 21]. Since NICCD if far more prevalent than type 1 tyrosinemia among East Asians, LF/MCT formula should always be attempted and high volume of intravenous fluids with glucose should be avoided in patients with liver decompensation. When there is a high suspicion of type 1 tyrosinemia, phenylalanine and tyrosine restricted formula without lactose should be a better option than overall protein restriction. Genetic testing results takes weeks or months, but polymerase chain reaction (PCR) sreening for hot-spot mutations in *SLC25A13* gene, serum pancreatic secretory trypsin inhibitor (PSTI), and blood/urine succinylacetone will differentiate most cases of NICCD from tyrosinemia type 1 within days.

Song et al. [13, 14] reported 3 male infants died from citrin deficiency. One had NICCD with compound heterozygous IVS6 + 5G > A/R319X mutation and died from central nervous system infection at the age of nine months. Another NICCD child with single heterozygous mutation died because of severe infection and disseminated intravascular coagulation (DIC). The third child with homozygous 851del4 mutation had liver cirrhosis, gross developmental delay, and dyslipidemia died from hepatic coagulopathy. Six out of eight NICCD children in our series had severe or recurrent infections before they die, and percentage of infection was significantly higher when compared to the survival group. Infections should be actively ruled out in NICCD children with liver decompensation, and aggressively treated if present.

Mean age and standard deviation at referral for the mortality group in our cohort was 9.58 ± 5.03 months, and significantly higher than that of the survival group (3.96 ± 3.13 months, *p*<0.000). Significantly more children in the mortality group never received LF/MCT formula or failed to comply with the change in feeding. Previous reports of mortality or liver transplant provided referral age as 7 months [11], and 3 months [10]. Late referral, atypical findings on serum amino acid profiles, and misdiagnosis (such as tyrosinemia type 1, or bile acid synthesis defect) may all have contributed to late referral or delayed switch to LF/MCT formula. In our practice with neonatal cholestasis, there was a shift towards changing all the formula diet to LF/MCT formula unless the infant was exclusively breast-fed and liver synthetic/metabolic parameters are normal. When there was liver decompensation, significant metabolic derangement, or non-response to conventional therapy, exclusively breastfeed infants with neonatal cholestasis should also be put on LF/MCT formula in order to prevent exacerbation until NICCD is ruled out. Or data and other reports suggested that suspected NICCD cases should be referred to tertiary centers as early as possible.

We previously reported an eight-month-old apparently healthy infant who developed acute liver failure and died few days before the scheduled liver transplant [18]. Genetic testing and mass spectrometry confirmed the diagnosis of citrin deficiency, but serum GGT levels were normal. We conducted detailed analyses of complete blood count and serum biochemistry profiles in our NICCD series. Lethal cases had significantly higher ammonia levels, while survived cases had significantly higher platelet count, and higher levels of GGT as well as total cholesterol. Significantly reduced platelet count in the mortality group may due to secondary thrombocytopenia from infection, hyper-splenism, or DIC. Thrombocytopenia is also associated in chronic liver diseases, especially in patients with liver failure [22]. Lower GGT, total cholesterol levels, and higher blood ammonia level maybe indications of impaired liver synthesis and clearance due to end stage liver diseases.

Guo et al. [15] presented one case of NICCD with gross developmental delay due to prolonged hepatosplenomegaly and recurrent ascites that progressed into lethal hepatic encephalopathy at his age of 22 months. Treepongkaruna et al. [16] from Thailand reported 2 NICCD cases that developed cirrhosis, one underwent liver transplant, another died of complications of end-stage liver disease. Chew et al. [17] reported a lethal case from Malaysia, neonatal cholestasis progressed to cirrhosis and the patient died from liver failure after sepsis at the age of 7 months. Serum amino acid profile was suggestive of citrin deficiency with marked elevation of methionine level and slight elevation of citrulline and tyrosine levels. However, absence of SLC25A13 gene mutation may suggest diagnosis other than NICCD.

In this report, 14.8% (9/61) NICCD patients died. This higher frequency of death, compared to other published reports, maybe attributed to a referral bias. NICCD is a recognized disease in major centers across China, and only patients with severe liver damage tend to be referred to our center. Other than that, previous reports of poor outcome were mostly reported by centers specialized with metabolic diseases; patients with severe liver diseases may have died before being diagnosed by metabolic specialists. Liver transplant may have helped these children to survive as indicated in previous reports [10–12, 16], but many factors surrounding the death (unexpected death, retrospective diagnosis, refusal to treatment, severe infection, death during local care, and diagnostic dilemma) may have prevented timely enlisting for transplantation. Morioka et al. reviewed large series of pediatric patients with various inheritable metabolic disorders received living donor liver transplant from parents who were heterozygous carriers of a gene mutation. None of these children experienced mortality and morbidity related due to heterozygous nature of the liver graft [23]. Parental origins of mutation should be determined for all cases, and heterozygous parents should be considered for living donor if NICCD infants need liver transplantation.

In order to prevent poor outcome in NICCD, we recommend the following strategies for screening, referral, diagnosis, and treatment in patients with neonatal cholestasis after excluding biliary atresia: (1) In primary and secondary care settings, any infant of East Asian origin with neonatal cholestasis should be screened for clues of NICCD (low birthweight, failure to thrive, hepatomegaly, fatty liver, hypoproteinemia, decreased coagulation factors, hemolytic anemia, and hypoglycemia). In infants of non-East Asian origin, NICCD screening may be prioritized after considering other more prevalent etiologies; (2) Suspected NICCD cases and cholestatic infants who did not respond to conventional treatment (ursodeoxycholic acid and fat soluble vitamins) should be referred to tertiary centers early on; (3) Tertiary care centers should consider changing to LF/MCT formula feeding even before the availability of metabolic and genetic screening results; (4) Any signs of infection should prompt adequate anti-microbial intervention; (5) When blood tyrosine is elevated but citruline is normal, still consider the possibility of NICCD until proven otherwise. Phenylalanine and tyrosine restricted formula without lactose should be a better option rather than overall protein restriction; (6) Genetic testing may take weeks or months before diagnosis, but polymerase chain reaction (PCR) screening for population specific hot-spot mutations in *SLC25A13* and *FAH* (fumarylacetoacetase) gene, serum pancreatic secretory trypsin inhibitor (PSTI), and blood/urine succinylacetone will be available within days, and should be considered for differential diagnosis of NICCD from tyrosinemia type 1. (7) In cases with hypoglycemia, frequent oral or gastric-tube feeding should be preferred rather than high volume intravenous glucose supplementation; (8) Patients with liver failure and low levels of platelet/GGT/ cholesterol or high levels of ammonia/tyrosine should be considered for early liver transplantation.

## Conclusion

We reported the largest series of NICCD mortality from a single center. This report is contrary to the traditional concept that NICCD is a benign process, and a significant proportion of patients in tertiary care setting may die or need liver transplant if diagnosis or treatment is delayed. Our data suggested that, late referral, delayed treatment with LF/MCT formula, low platelet count, low levels of GGT, total cholesterol, blood citrulline, and high level of blood ammonia and tyrosine, were all associated with poor prognosis. We also suggested strategies for preventing poor outcome in NICCD.

## Additional file

Additional file 1: Original data used for statistical analyses. (XLS 41 kb)

## Abbreviations

AFP: Alpha-fetoprotein
ALT: Alanine aminotransferase
APTT: Activated partial thromboplastin time
AST: Aspartate aminotranferase
BASD: Bile acid synthesis defect
CTLN2: Citrullinemia type II
Fib: Fibrinogen
FTTDCD: Failure to thrive and dyslipidemia caused by citrin deficiency
GGT: Gamma-glutamyl transpeptidase
INR: International normalized ratio
LF/MCT formula: Lactose-free and/or medium-chain triglycerides enriched formula
MCT: Medium chain triglycerides
NICCD: Neonatal intrahepatic cholestasis caused by citrin deficiency
PFIC1: Progressive familial intrahepatic cholestasis type 1
PLT: Blood platelet count
PT: Prothrombin time
PTA: Prothrombin activity
RBC: Red blood cell count
SLC25A13: Solute carrier family 25 member 13
TBA: Total bile acid
TCH: Total cholesterol
TT: Thrombin time
UDCA: Ursodeoxycholic acid
WBC: White blood cell count

## References

[1] Kobayashi K, Saheki T, Song YZ. Citrin Deficiency. 2005 Sep 16 [Updated 2014 Jul 31]. In: Pagon RA, Adam MP, Ardinger HH, et al., editors. GeneReviews® [Internet]. Seattle (WA): University of Washington, Seattle; 1993–2016.

[2] Kobayashi K, Sinasac DS, Iijima M, Boright AP, Begum L, Lee JR, Yasuda T, Ikeda S, Hirano R, Terazono H, Crackower MA, Kondo I, Tsui LC, Scherer SW, Saheki T (1999). The gene mutated in adult-onset type II citrullinaemia encodes a putative mitochondrial carrier protein. Nat Genet.

[3] Dimmock D, Maranda B, Dionisi-Vici C, Wang J, Kleppe S, Fiermonte G, Bai R, Hainline B, Hamosh A, O'Brien WE, Scaglia F, Wong LJ (2009). Citrin deficiency, a perplexing global disorder. Mol Genet Metab.

[4] Saheki T, Kobayashi K (2002). Mitochondrial aspartate glutamate carrier (citrin) deficiency as the cause of adult-onset type II citrullinemia (CTLN2) and idiopathic neonatal hepatitis (NICCD). J Hum Genet.

[5] Tabata A, Sheng JS, Ushikai M, Song YZ, Gao HZ, Lu YB, Okumura F, Iijima M, Mutoh K, Kishida S, Saheki T, Kobayashi K (2008). Identification of 13 novel mutations including a retrotransposal insertion in SLC25A13 gene and frequency of 30 mutations found in patients with citrin deficiency. J Hum Genet.

[6] Lu YB, Kobayashi K, Ushikai M, Tabata A, Iijima M, Li MX, Lei L, Kawabe K, Taura S, Yang Y, Liu TT, Chiang SH, Hsiao KJ, Lau YL, Tsui LC, Lee DH, Saheki T (2005). Frequency and distribution in East Asia of 12 mutations identified in the SLC25A13 gene of Japanese patients with citrin deficiency. J Hum Genet.

[7] Chen R, Wang XH, Fu HY, Zhang SR, Abudouxikuer K, Saheki T, Wang JS (2013). Different regional distribution of SLC25A13 mutations in Chinese patients with neonatal intrahepatic cholestasis. World J Gastroenterol.

[8] Shigematsu Y, Hirano S, Hata I, Tanaka Y, Sudo M, Sakura N, Tajima T, Yamaguchi S (2002). Newborn mass screening and selective screening using electrospray tandem mass spectrometry in Japan. J Chromatogr B Analyt Technol Biomed Life Sci.

[9] Hicks J, Chen HL, Jeng Y, Chang MH (2012). Neonatal intrahepatic cholestasis associated with Citrin Deficiency (NICCD). Lab Investig.

[10] Ohura T, Kobayashi K, Tazawa Y, Abukawa D, Sakamoto O, Tsuchiya S, Saheki T (2007). Clinical pictures of 75 patients with neonatal intrahepatic cholestasis caused by citrin deficiency (NICCD). J Inherit Metab Dis.

[11] Tamamori A, Okano Y, Ozaki H, Fujimoto A, Kajiwara M, Fukuda K, Kobayashi K, Saheki T, Tagami Y, Yamano T (2002). Neonatal intrahepatic cholestasis caused by citrin deficiency: severe hepatic dysfunction in an infant requiring liver transplantation. Eur J Pediatr.

[12] Shigeta T, Kasahara M, Kimura T, Fukuda A, Sasaki K, Arai K, Nakagawa A, Nakagawa S, Kobayashi K, Soneda S, Kitagawa H (2010). Liver transplantation for an infant with neonatal intrahepatic cholestasis caused by citrin deficiency using heterozygote living donor. Pediatr Transplant.

[13] Song YZ, Deng M, Chen FP, Wen F, Guo L, Cao SL, Gong J, Xu H, Jiang GY, Zhong L, Kobayashi K, Saheki T, Wang ZN (2011). Genotypic and phenotypic features of citrin deficiency: five-year experience in a Chinese pediatric center. Int J Mol Med.

[14] Song YZ, Li BX, Chen FP, Liu SR, Sheng JS, Ushikai M, Zhang CH, Zhang T, Wang ZN, Kobayashi K, Saheki T, Zheng XY (2009). Neonatal intrahepatic cholestasis caused by citrin deficiency: clinical and laboratory investigation of 13 subjects in mainland of China. Dig Liver Dis.

[15] Guo L, Li BX, Deng M, Wen F, Jiang JH, Tan YQ, Song YZ, Liu ZH, Zhang CH, Kobayashi K, Wang ZN (2011). Etiological analysis of neurodevelopmental disabilities: single-center eight-year clinical experience in South China. J Biomed Biotechnol.

[16] Treepongkaruna S, Jitraruch S, Kodcharin P, Charoenpipop D, Suwannarat P, Pienvichit P, Kobayashi K, Wattanasirichaigoon D (2012). Neonatal intrahepatic cholestasis caused by citrin deficiency: prevalence and SLC25A13 mutations among Thai infants. BMC Gastroenterol.

[17] Chew HB, Ngu LH, Zabedah MY, Keng WT, Balasubramaniam S, Hanifah MJ, Kobayashi K (2010). Neonatal intrahepatic cholestasis associated with citrin deficiency (NICCD): a case series of 11 Malaysian patients. J Inherit Metab Dis.

[18] Zhang MH, Gong JY, Wang JS (2015). Citrin deficiency presenting as acute liver failure in an eight-month-old infant. World J Gastroenterol.

[19] Fu HY, Zhang SR, Wang XH, Saheki T, Kobayashi K, Wang JS (2011). The mutation spectrum of the SLC25A13 gene in Chinese infants with intrahepatic cholestasis and aminoacidemia. J Gastroenterol.

[20] Saheki T, Inoue K, Ono H, Katsura N, Yokogawa M, Yoshidumi Y, Furuie S, Kuroda E, Ushikai M, Asakawa A, Inui A, Eto K, Kadowaki T, Sinasac DS, Yamamura K, Kobayashi K (2012). Effects of supplementation on food intake, body weight and hepatic metabolites in the citrin/mitochondrial glycerol-3-phosphate dehydrogenase double-knockout mouse model of human citrin deficiency. Mol Genet Metab.

[21] Saheki T, Kobayashi K, Terashi M, Ohura T, Yanagawa Y, Okano Y, Hattori T, Fujimoto H, Mutoh K, Kizaki Z, Inui A (2008). Reduced carbohydrate intake in citrin-deficient subjects. J Inherit Metab Dis.

[22] Giannini EG, Peck-Radosavljevic M (2015). Platelet dysfunction: status of Thrombopoietin in thrombocytopenia associated with chronic liver failure. Semin Thromb Hemost.

[23] Morioka D, Kasahara M, Takada Y (2005). Current role of liver transplantation for the treatment of urea cycle disorders: a review of the worldwide English literature and 13 cases at Kyoto University. Liver Transpl.

